# Proteomic analysis of drought stress response mechanism in soybean (*Glycine max* L.) leaves

**DOI:** 10.1002/fsn3.2168

**Published:** 2021-02-07

**Authors:** Seyed Hamid Yahoueian, Mohammad Reza Bihamta, Hamid Reza Babaei, Mitra Mohammadi Bazargani

**Affiliations:** ^1^ Department of Plant Breeding and Biotechnology, Science and Research Branch Islamic Azad University Tehran Iran; ^2^ University of Tehran Tehran Iran; ^3^ Horticulture Crops Research Department Khorasan Razavi Agricultural and Natural Resources Research and Education Center AREEO Mashhad Iran; ^4^ Agriculture institute Iranian Research Organization of Science and Technology Tehran Iran

**Keywords:** 2‐ DE, drought tolerance, leaf proteome, nano‐LC–MS/MS, soybean

## Abstract

Knowledge of the physiological and molecular mechanisms of drought responses is fundamental for developing genetically drought tolerant and high yielding crops. To understand molecular mechanism of drought tolerance of soybean (*Glycine max* L.), we compared leaf proteome patterns of in two genotypes GN‐3074 (drought tolerant) and GN‐2032 (drought‐sensitive) under drought stress during vegetative stage. Proteins were extracted from leaves of well‐watered and drought‐treated plants by using the trichloroacetic acid (TCA)–acetone precipitation method and analyzed by two‐dimensional polyacrylamide gel electrophoresis. Out 488 reproducibly detected and analyzed on two‐dimensional electrophoresis gels, 26 proteins showed significant changes in at least one genotype. The identification of 20 differentially expressed proteins using mass spectrometry revealed a coordinated expression of proteins involved in cellular metabolisms including photosynthesis, oxidative stress defense, respiration, metabolism process, signal transduction, phosphorus transduction, and methyl transduction which enable plant to cope with drought conditions. The most identified proteins involved in photosynthesis and oxidative stress defense system. The up‐regulation of several photosynthetic proteins and also high abundance of oxidative stress defense proteins in GN‐3074 genotypes as compare to GN‐2032 genotypes might reflect the fact that drought tolerance of GN‐3074 is due to effective photosynthetic machinery and more defense against oxidative stress. Our results suggest that soybean plant might response to drought stress by applying efficiently stay‐green mechanism through coordinated gene expression during vegetative stage.

## Graphical abstract

A coordinated expression of proteins involved in cellular metabolisms including photosynthesis, oxidative stress defense, respiration, metabolism process, signal transduction, phosphorus transduction, and methyl transduction which enable plant to cope with drought conditions. The up‐regulation of several photosynthetic proteins and also high abundance of oxidative stress defense proteins in tolerant genotype as compare to sensitive might reflect the fact that drought tolerance is due to effective photosynthetic machinery and more defense against oxidative stress.

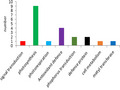

## 1 INTRODUCTION

The crop growth and development are constantly influenced by harsh environmental conditions which are the most important yield‐reducing factors in the world. Drought stress has been recognized as one of the crop performances limiting factors and a threat for successful crop production (Maleki et al., 2013), But acclimation to drought conditions is dependent upon the activation of a series of integrated processes including stress‐signal perception, signal transduction, gene and protein expression, and biochemical response at the cellular level (Chaves et al., 2003). In the physiological processes, drought severity stress‐induced several metabolic substances, which free proline has been just one of them. Proline acts as reservoir nitrogen and also as an osmotic potential reducer which helps plants to tolerate stresses. Stomata sensitivity to water deficit is a part of the principal resistance components, which its increasing causes resistance. Drought and heat decrease photosynthesis, stomata conductivity, and transpiration rate and also caused a decrease in the Co_2_ accumulation in leaves, Therefore, high stomata conductivity in plants is an advantage of water economy and drought resistance (Das et al., 2016). Drought stress plays an important role in physiological processes, metabolism, and expression of numerous genes which have a role in plant adaptation to water deficit stress. Proteomics has been identified as the most directed approach to relate the function of genes to the associated products. Applications of proteomics in identifying drought stress‐related proteins have been reported in several researches such as rice (*Oryza sativa* L.) (Salekdeh et al., 2002), Sunflower *(Heliantus annus*) (Ghaffari et al., 2017), common bean (*Phaseolus vulgaris* L.) (Gebeyehu et al. (2010), barley (*Hordeum vulgare* L.) (Rollins et al., 2013), soybean (*Glycine max* L.) (Yu et al., 2016). Soybean is one of the important plants that affect the environment (Luo et al., 2005). Drought tolerance is a complicated quantitative trait. Therefore, it is necessary to study plant response mechanisms in molecular aspects such as transcription, translation, and drought response metabolism. Many proteins modify their expression in response to drought and osmotic stresses (Zang & Komatsu, 2007). Details of molecular mechanisms regulating responses of plant genes to water stress remain to be discovered, and there are numerous questions to be considered at the molecular level. However, responses to drought are specific species and often specific genotypes (De Leonardis et al., 2007). Moreover, drought response of plants is influenced by the duration and severity of water loss (Pinheiro & Chaves, 2010), the age and also, stage of development at the point of drought exposure (De Leonardis et al., 2007), as well as the organ and cell type experiencing water deficits (Pastori & Foyer, 2002) but proteins are the primary molecules that carry out various biological functions in cells and entire organism. Alterations in proteome composition provide the basis for a plant to perform different biological functions, including adapting to changing and/or sub‐optimal environmental conditions (Yu et al., 2016). Alam has identified novel proteins such as a translation initiation factor, apyrase, auxin‐amidohydrolase, and coproporphyrinogen oxidase in soybean under response to waterlogging stress (Alam, Lee, et al., 2010). Also, Proteome analysis of soybean root under drought condition showed that two key enzymes involved in carbohydrate metabolism, UDP glucose pyrophosphorylase, and 2,3‐bisphosphoglycerate independent phosphor‐glycerate mutase, were down‐regulated upon exposure to drought (Alam, Sharmin, et al., 2010). Castillego et al. (2008) observed a general decrease in proteins expression corresponding to photosynthesis enzymes and carbohydrate metabolism in susceptible sunflower genotype under drought stress, suggesting inhibition of the energetic metabolism, whereas, similar these changes have not been observed in the tolerant genotype, indicating a normal metabolism under drought stress (Castillejo et al., 2008). The mechanism responsive to stress in various soybean tissues depends upon the severity, duration, and type of stress that led to various changes at the proteome level. The nature and intensity of responses may vary depending on the stress (Hossain et al., 2013). Therefore, the proteome responses to drought stress and understanding the drought stress mechanism effect on cellular process of soybean. In the present study, we aimed to identify molecular mechanism of drought stress response at proteome level in two contrasting soybean genotypes differing in responses to drought. Proteomics approaches are favorable to characterize the responses of plants exposed to water deficiency. Accordingly, in the present study, physiology and proteomics techniques were used to examine the response of soybean genotypes to drought conditions. Although there is a shortage in proteomic studies of contrasting soybean genotypes, it is important that such studies be conducted to determine proteins and identified molecular mechanisms in response to drought stress in soybean, which would help accelerate its genetic improvement.

## 2 MATERIALS AND METHODS

### 2.1 Plant material and physiological evaluation

The experiments were carried out over 2 years (2016–2017) in the research station of the oilseeds, seed, and plant improvement institute, Karaj. Iran. Two genotypes were selected throughout 10 genotypes during field experiments, in the first year. The experimental design was factorial with three replications in greenhouse in second year. The factorial treatments included combinations of two water regimes, well‐watered and drought, and two soybean genotypes GN‐2032 (drought‐sensitive) and GN‐3074 (drought tolerant) which showed significant differences in tolerance under drought stress in previous studies during 2013–2016. The sowing date was 2 May 2016. The dimensions of the Pots were 19 × 13 × 19 cm. The pots were filled with field soil. The soil humidity was measured by TDR Model 6050 × 1. Plants were grown in a temperature‐controlled greenhouse under 22/16°C day/night cycle and under well‐watered conditions until V_4_ stage. Drought treatment was initiated at V_4_ Stage; the soil moisture was maintained at 15% and 50% soil moisture capacity in well‐watered and drought conditions, respectively. Drought treatment was exposed for three weeks, while control plants remained well‐watered (normal moisture conditions, 85%) during the period of the experiment.

Leaf samples were taken from both stressed plants and well‐watered controls during V4 stage once every week (three times: 7, 14, and 21 days after stress). The sampled leaf was the last fully growth leaf which located on the last fourth stem node. Therefore, in every time of sampling, the sampled leaves were in same ages, physiologically. The proline content, stomata conductivity was measured in last fully growth leaf, according to Bates (1973). Stomata conductivity measured by using a promoter, model AP4, during the 9–10 A.m. All the collected samples for proteomics analysis were frozen in liquid nitrogen immediately and stored at −80°C.

### 2.2 Protein extraction

After physiological data analysis and identifying intended sample which revealed the most difference, fresh leave materials (500 mg) were ground to powder in liquid nitrogen with a mortar and pestle for selected drought treatment and control in all the three replications. The powder was transferred to 10% trichloroacetic acid (TCA) solution with 0.07% 2‐mercaptoethanol in acetone. The mixture was vortexed and then sonicated for 5 min, then incubated for 1 hr at −20°C. After incubation, the suspension was centrifuged at 9000 *g* for 20 min at 48°C. The supernatant was discarded, and the resulting pellet was washed twice with 0.07% 2‐mercaptoethanol in acetone. The resulting pellet was dried by using a Speed‐Vacuum concentrator (Savant Instruments) and resuspended with 8 M urea, 2 M thiourea, 5% CHAPS, and 2 mM tributyl phosphine by vortex for 1 hr at 258°C. The suspension was centrifuged at 20,000 *g* for 20 min at 258°C. Supernatant was collected as a protein extract.

### 2.3 Two‐dimensional polyacrylamide gel electrophoresis

Isoelectric Focusing (IEF) was carried out on immobilized pH gradient (IPG) strips (17cm, pH 4–7 L) (Bio‐Rad). The running condition was as follows: First using a gradually increasing voltage (150V–3500 V) and then reaching 42,000 V/h during 4 steps and 16 hr (Zamani, 2007).

Focused strips were equilibrated for 15 min in 6–7 ml equilibration solution containing 6 M urea, 30% (w/v) glycerol, 2% (w/v) SDS, 1% (w/v) DTT, and 50 mM Tris–HCl buffer, pH 8.8. Separation of proteins in the second dimension was performed by SDS‐PAGE in a vertical slab of acrylamide (13% total monomer, with 2.6% cross‐linker) using a Dodeca Cell (Bio‐Rad) and electrophoresis run at 50 mA/gel until the Dye front reached the bottom of the gel. For analytical and preparative gels, 120 and 800 μg protein were loaded, respectively. The protein spots in analytical and preparative gels were visualized by silver nitrate and coomassie brilliant blue (CBB G‐250), respectively (Zamani, 2007).

### 2.4 Gel images analysis

Analytical gels were scanned at a resolution of 600 dots per inch with a GS‐ 800 imaging densitometer (Bio‐Rad). The Melanie 4 software was used to analyze gels and compare them (GeneBio). Gel analysis included spot detection, protein quantification, and spot pairing which were carried out based on Melanie 4 default settings, and spot pairs were investigated visually. The molecular masses of the proteins on the gels were determined by coelectrophoresis of standard protein markers (GE Healthcare), and the pI of the proteins was determined from the distance that spots migrated on IPG strips (17 cm, pH 4e 7 L). One 2‐DE gel was run per plant for three independent biological replicates and the percentage volume of each protein spot was estimated and analyzed. ANOVA was conducted by SAS 9.2 software and means were compared with the LSD test at *p* < .05. Spots were only considered to be significantly up‐ or down‐regulated at *p* < .05. Impact Factors (IF) were calculated by dividing the percentage volume of spots on gels in drought stress by the percentage volume of spots in control samples. Repeatable spots were selected which showed significant changes to drought stress and their IF was more than 1.5 or less than 0.66 (CAO et al., 2015) and then samples were loaded on CBB Gel and identified spots were sliced from CBB Gel. The sliced spots were stored in liquid nitrogen and identified by nano‐LC–MS/MS against the NCBI protein database.

### 2.5 Peptide preparation for mass spectrometry analysis

Identify of proteins in protein spots by mass spectrometer (Ramseur et al., 2004), protein spots were excised from Comassie 2‐DE gel and distained in 50 mM ammonium bicarbonate for 1 hr at 408°C. Proteins in the excised gel pieces were reduced by incubation in 10 mM dithiothreitol in 100 mM NH_4_HCO_3_ for 1 hr at 608°C, followed by incubation for 30 min with 40 mM iodoacetamide in 100 mM NH4HCO3. The gel pieces were minced and allowed to dry then rehydrated overnight at 37°C in 100 mM NH_4_HCO_3_ containing 1 pM trypsin (Wako). The resulting tryptic peptides were extracted from the gel grains three times with 0.1% trifluoroacetic acid in 50% acetonitrile. The procedure was performed with DigestPro (Intavis Bioanalytical Instruments). The final peptide solution was dried and then reconstituted with 30 ml 0.1% trifluoroacetic acid in 5% acetonitrile and desalted with NuTip C‐18 pipet tips (Glygen). The desalted peptide solution was analyzed by nano‐liquid chromatography (LC)–tandem MS/MS.

### 2.6 Protein identification by nano‐LC–MS/MS

A nanospray LTQ XL Orbitrap MS (ThermoFisher Scientific) was operated in data‐dependent acquisition mode with the installed XCalibur software (ThermoFisher Scientific). Peptides in 0.1% formic acid were loaded onto a C18 Pep Map trap column (300 mm ID by 5 mm; Thermo Fisher Scientific), using UltiMate 3000 nano‐liquid chromatography (Dionex). The peptides were eluted from the trap column, and their separation and spraying were done on a 3‐mm nano‐capillary column, 75 mm ID by 15 cm (NTTC‐ 360/75‐3; Nikkyo Technos) with 0.1% formic acid in acetonitrile at a flow rate of 200 nl min‐1. Samples were sprayed into the mass spectrometer by using a PicoTip emitter (20 mm ID, 10 mm tip ID; New Objective) at a spray voltage of 1.8 kV. Full‐scan mass spectra were acquired in the orbitrap over a mass range of 150–200 m/z (mass: charge) with a resolution of 15,000. The three most intense ions above an intensity threshold of 1,000 units were selected for collision‐induced fragmentation in the linear ion trap at a normalized collision energy of 35% after accumulation to a target value of 1,000 intensity units. Dynamic exclusion was employed within 30 s to prevent repetitive selection of peptides. Acquired MS/ MS spectra were converted to individual DTA files by using BioWorks software (version 3.3.1; Thermo Fisher Scientific). The following parameters were set to create a peak list: parent ions in the mass range with no limitation, one grouping of MS/MS scans, and threshold of 100. The resulting peptide sequence data were used to search the NCBInr protein database via the Mascot search engine (version 2.2.04; Matrix Science). Flowering plants were selected as the Taxonomy parameter, and carbamidomethylation of cysteines and oxidation of methionine were set as the fixed and variable modifications, respectively. Trypsin was specified as the proteolytic enzyme and one missed cleavage was allowed. The search parameters were peptide mass tolerance 10 ppm; fragment mass tolerance 0.2 Da; maximum missed cleavages 1; and peptide and charges +1, +2, and + 3. A homology search of the amino acid sequences of identified proteins was performed against the NCBI non‐redundant sequence database by using BLASTP to assign protein identities.

## 3 RESULTS AND DISCUSSION

### 3.1 Effect of drought stress on physiological traits of soybean

**FIGURE 1:**
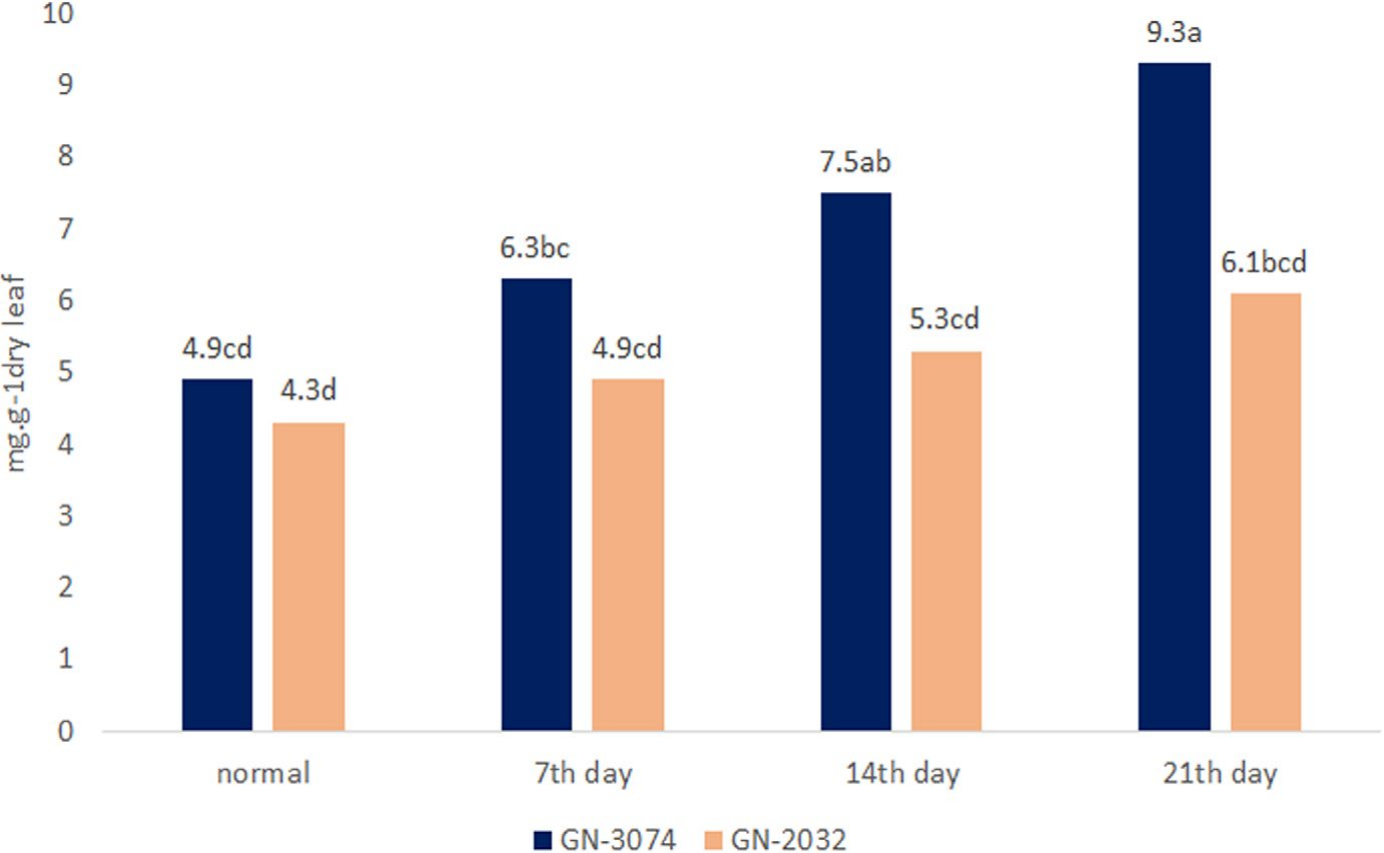
Comparisons of proline means during the drought stress by Duncan 5% level. The numbers have similar letters, are non‐significant and on same level

Different physiological characteristics were affected differentially by drought stress. Proline content was induced, after drought treatment and increased significantly as drought stress continued. It was found to be higher in genotype GN‐3074 than genotype GN‐2032 (Figure 1). It is noteworthy that higher proline content was observed in genotype GN‐3074 than genotype GN‐2032 under normal condition, too. From the seventh day, proline increased more intensely in the tolerant genotype, while it was very low in sensitive genotype. The highest proline difference between the two genotypes was observed on 21th (8.91 vs. 6.124).

**FIGURE 2:**
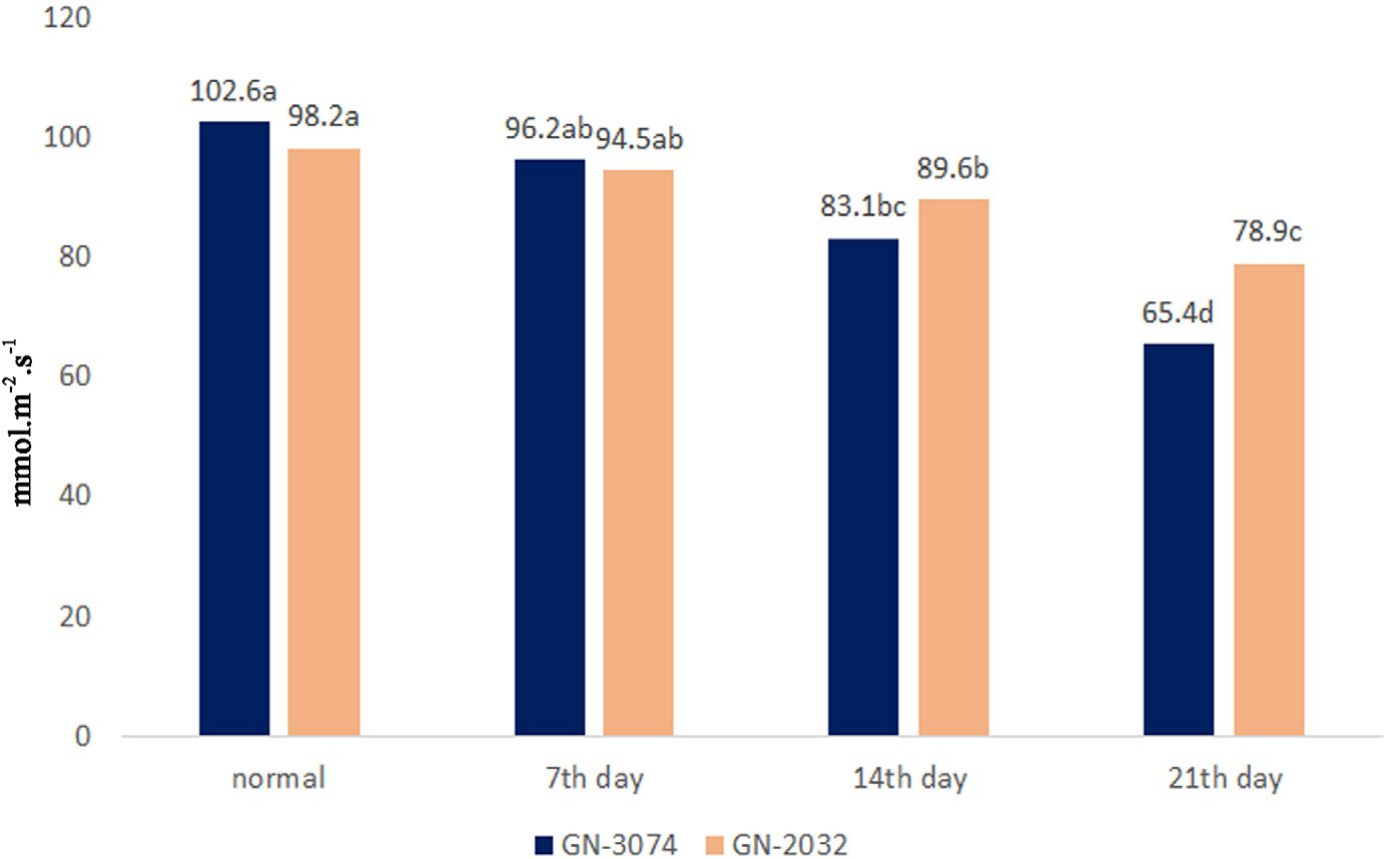
Comparison of stomata conductivity averages by Duncan in 5% level. The numbers have similar letters, are non‐significant and on same level

The variation in stomatal behavior among extant plant groups has stimulated great interest recently across diverse fields of science. Behavioral differences in the responses of stomata to water stress within plant communities have been recognized as an important axis of variation in ecological strategy (Martínez‐Vilalta & Garcia‐Forner, 2017). Stomatal closing view as a drought tolerance mechanism to avoid excess water loss via transpiration. Water deficiency reduced Stomata conductivity in the GN‐3074, but its severity was less than the GN‐2032. The highest stomata conductivity was related to the GN‐3074 and under normal condition (102.6 mmol.m^‐2^.S^‐1^). (Figure 2). Decrease in stomatal conductance is caused by the reduction of photosynthesis, electron transport, and photophosphorylation. These can affect reduction in ATP synthesis which is an initial response to water deficits, can lead to reduction of the capacity for Ribulose‐1,5‐bisphosphate (RuBP) regeneration, and finally, it can reduce potential photosynthesis (Lawlor & Tezara, 2009). Furthermore, Rubisco activity may be impaired by Rubisco Activase activity and the reduction of ATP. Inhibitors such as RuBP analogs bind to the active site of Rubisco, decreasing its activity, especially when the concentration of RuBP is under‐saturated due to water deficiency. The regulation and restoration of Rubisco are mediated by Rubisco Activase and require a high rate of ATP/ADP conversion. Thus, because phosphorylation is reduced under water stress, the activity of Rubisco is depressed (Parry et al., 2002).

### 3.2 Proteomic changes in soybean leaves following drought stress

**FIGURE 3:**
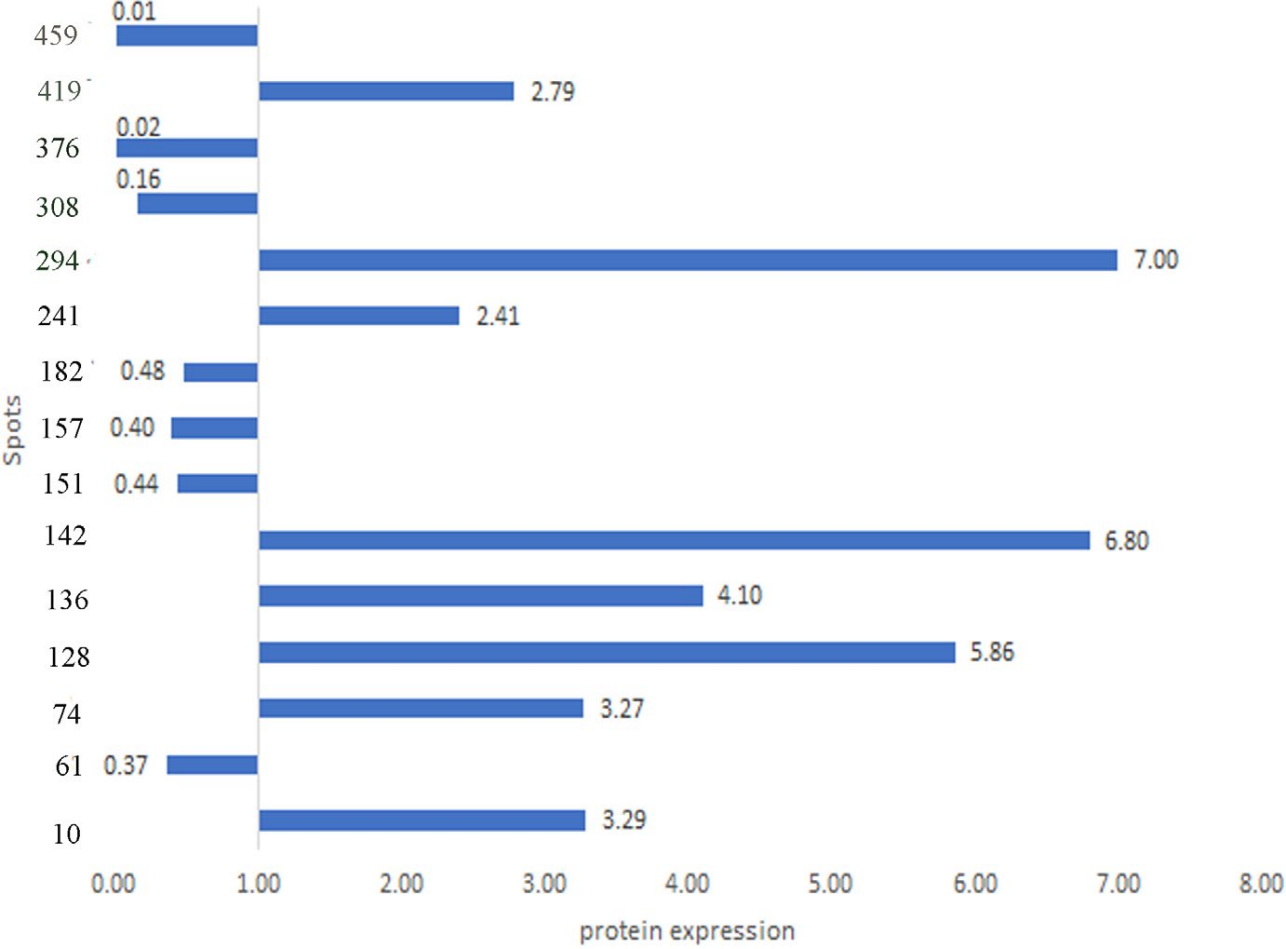
Proteins expressions on silver nitrate gels in GN‐3074 (tolerant). Numbers on the bars denote the spots numbers on the gels. Right bars indicate up‐regulating and left bars down‐regulating. Numbers on the bars indicate IFs

**FIGURE 4:**
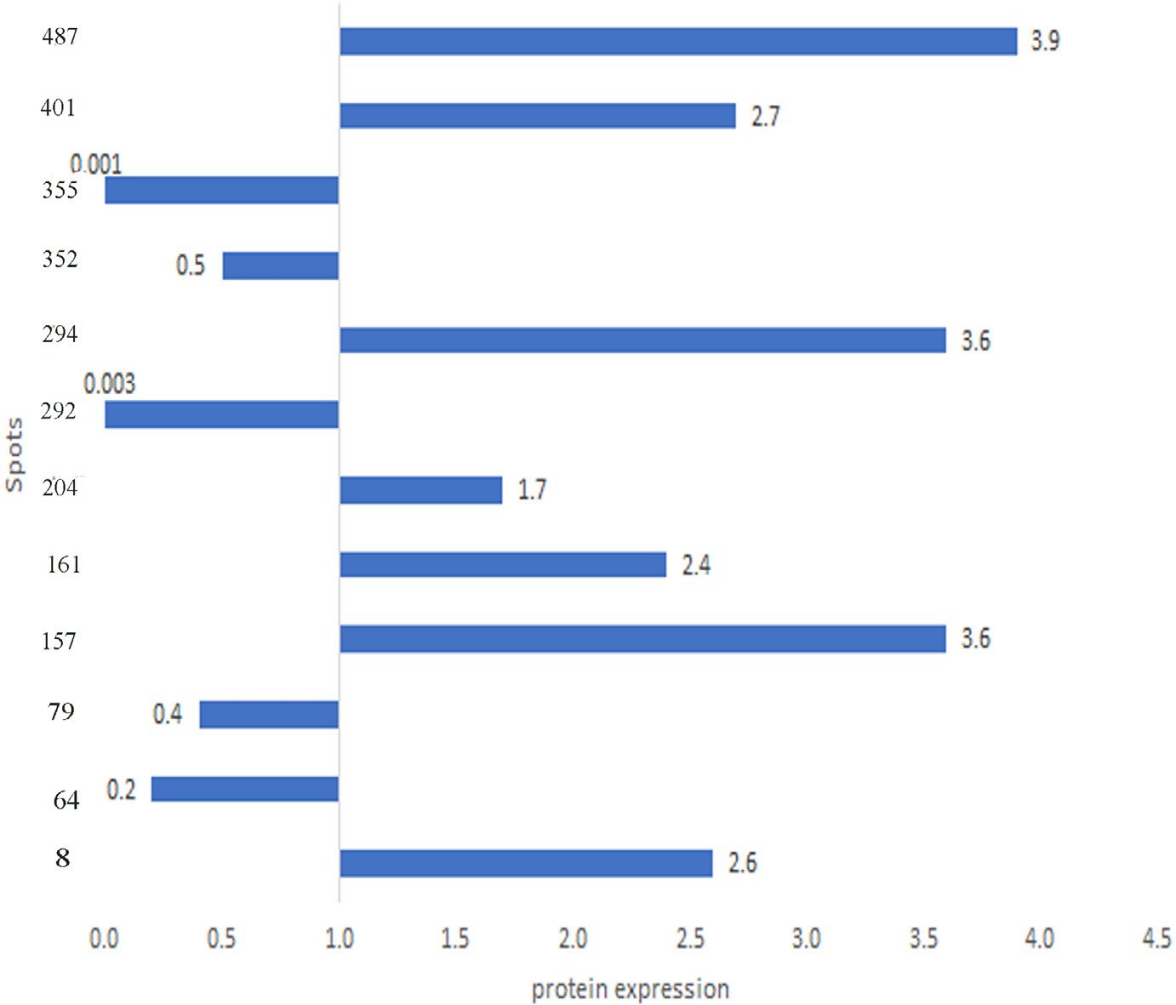
Proteins expressions on silver nitrate gels in GN‐2032 (sensitive). Numbers denote the spots numbers on the gels. Right bars indicate up‐regulating and left bars down‐regulating. Numbers on the bars indicate IFs

**FIGURE 5:**
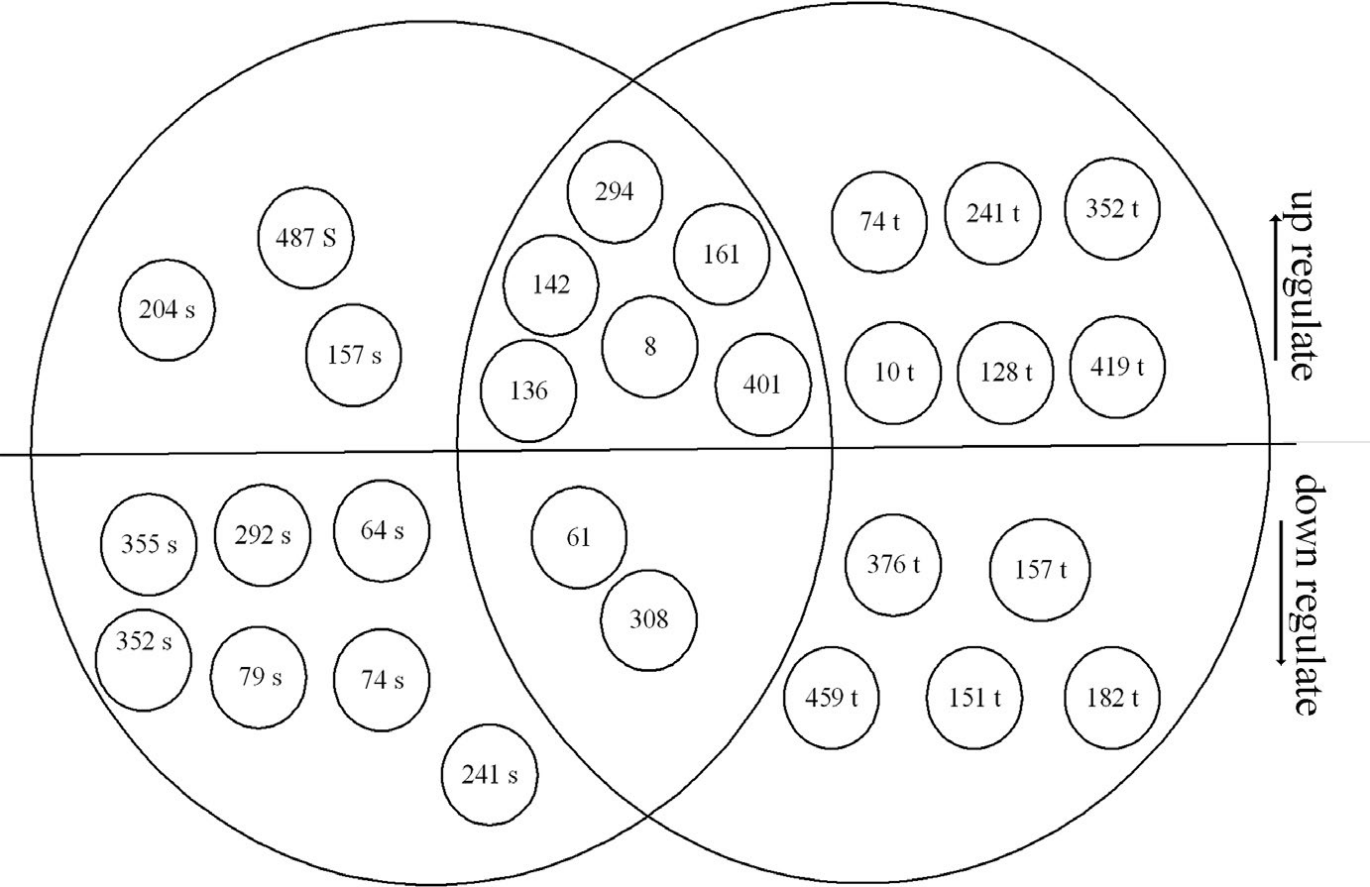
Venn diagram analysis of common drought‐responsive proteins in the leaves of sensitive (GN‐2032) and tolerant (GN‐3074) soybean genotypes. Overlapping proteins denote common protein spots between the genotypes. Numbers correspond to the protein spots in two‐dimensional polyacrylamide gel electrophoresis patterns. Letters denote the proteins from sensitive (s) and tolerant (t) genotype. Arrows indicate increase (↑) and decrease (↓) in abundance of related proteins

Proteomic patterns of leaves in drought‐stress‐treated and control plants were compared by 2‐DE. Comparison of 2‐DE gels by using Melanie 4 software revealed 488 reproducible protein spots. Obtained results showed that drought could have significant effects on 280 spots; among them, we eliminated spots that had CV% of over 20% as well as those whose IFs were higher than 1.5 or lower than 0.6. A total of 14 spots remained in GN3074 (tolerant), of which seven spots were up‐regulated and seven were down‐regulated under drought condition. The 12 remained spots in GN‐2032 (sensitive) included 6 up‐regulated and 6 down‐regulated spots (Figures 3, 4, 5).

### 3.3 Identified Proteins

**FIGURE 6:**
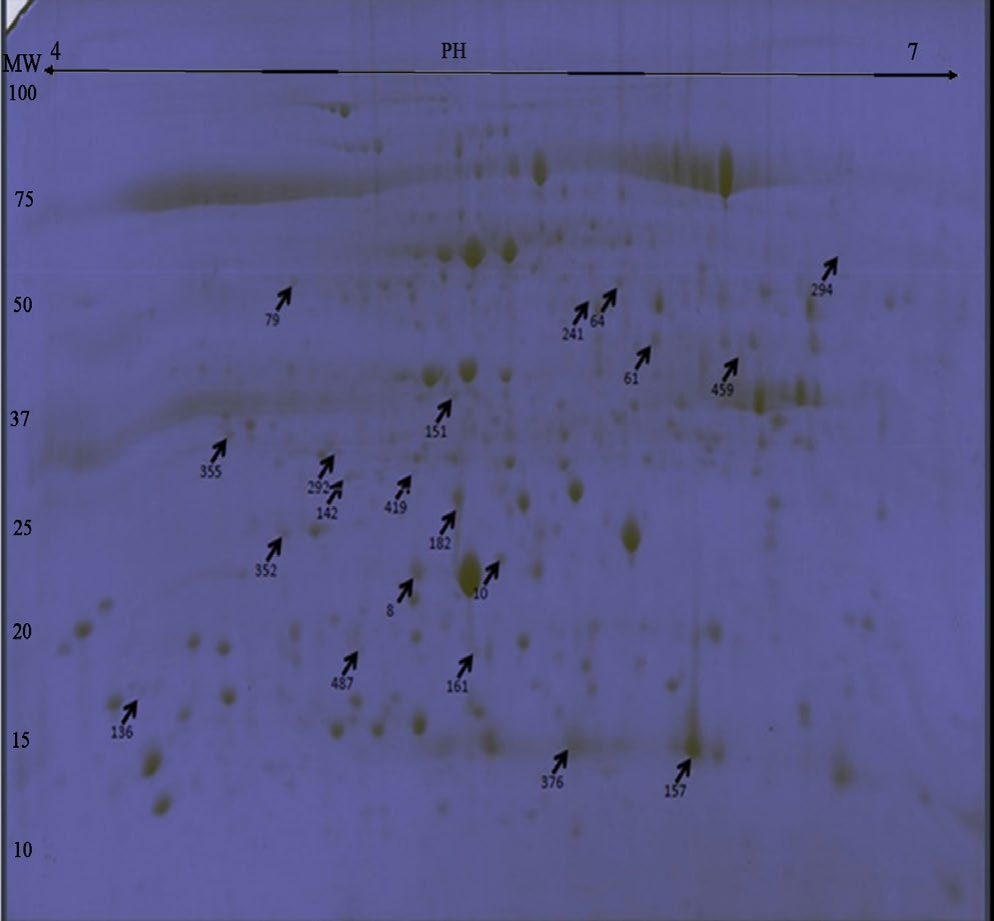
Two‐dimensional Comassie Brilliant Blue gel electrophoresis (2‐DE) and Sliced Spots on Comassie Blue Gel (CBB GEL). After spots selections by 2 DE gels stained in silver nitrate, Proteins were extracted from the both genotypes leave at the end of drought treatment, were mixed each other and separated by 2‐DE, and stained with Coomassie Brilliant Blue. Arrows and numbers indicate final selected proteins situations

**FIGURE 7:**
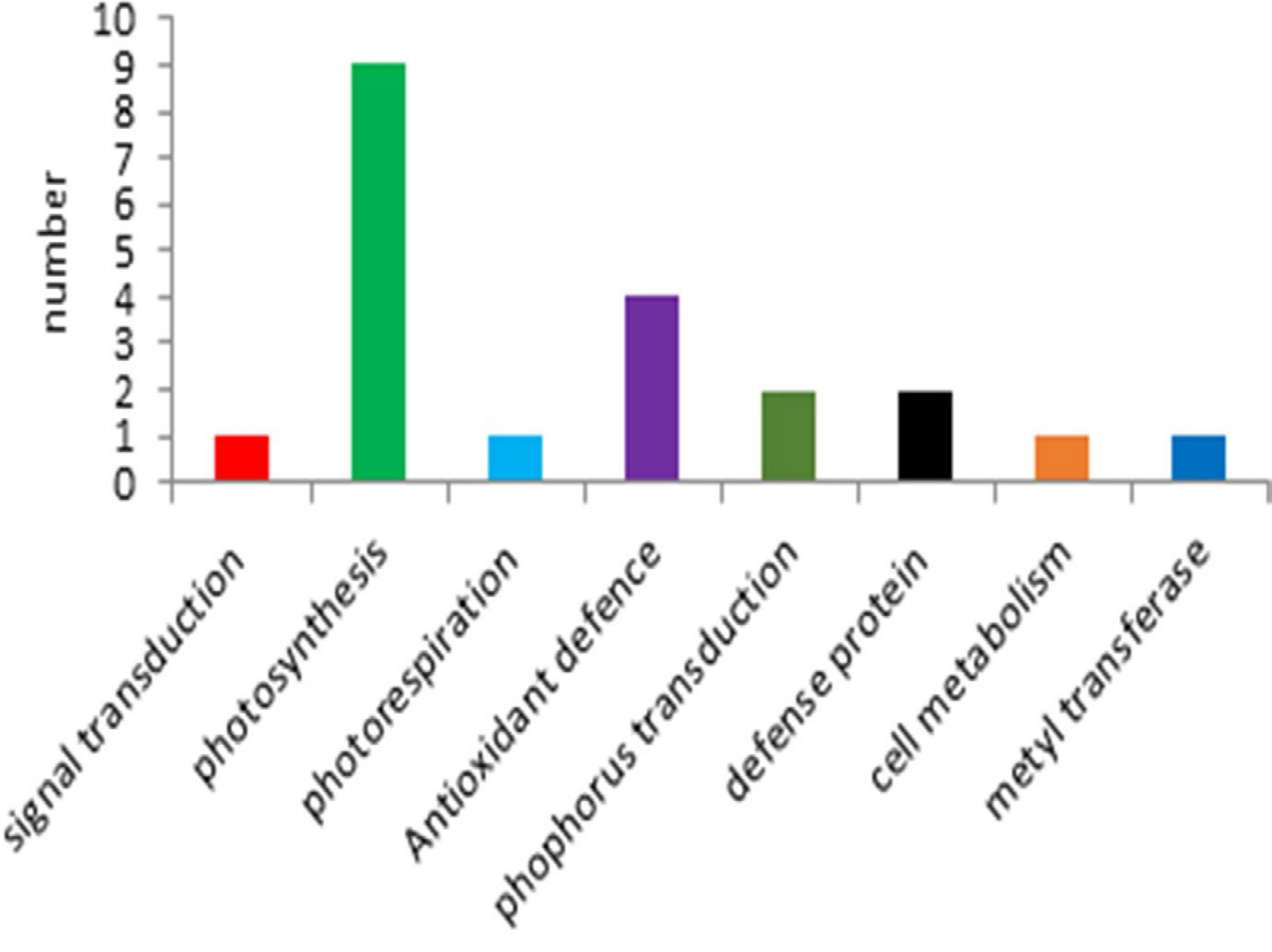
Grouping the functions of identifying protein in soybean leaves. According to the direction of changes, all identified proteins were categorized into eight groups

A total of 20 spots were identified on CBB Gel (Figure 6). Identified proteins were assigned to functional groups. According to the direction of changes, all identified proteins were categorized into several groups. The majority of the selected proteins were related to photosynthesis, which included six proteins. Subsequently, the proteins involved in defense mechanisms were found to be the most abundant. Other proteins were involved in photorespiration, respiration, metabolism process, signal transduction, phosphorus transduction, and Methyl transduction (Figure 7).

Drought stress was able to affect photosynthesis and also activate the anti‐oxidant defense system. The most identified proteins were involved in photosynthesis, including ferredoxin‐NDP reductase, chlorophyll a‐b binding protein of LHCII, RUBISCO Activase, chlorophyll a–b binding protein, RUBISCO, and Ribolus bisposphate carboxylase small chain were involved in the three levels of photosynthesis such as light‐harvesting, electron transport chain, and the Calvin cycle. (Tables 1 and 2, Figure 7). Out of six proteins involved in photosynthesis, two proteins, Chlorophyll a–b binding protein (spot 142) and Ribolus bisposphate carboxylase small chain PW9 (spot 419) were up regulated in both genotypes, with the difference that they had obviously upregulation up to three and seven folds in tolerant genotype GN‐3074 under drought stress, furthermore four other protein had decrease in abundance or were absent in sensitive genotype GN‐2032 while they didn't show any significant change or up‐regulated in tolerant genotype GN‐3074 under drought stress (Tables 1 and 2). One of the Rubisco‐related proteins is Rubisco Activas which plays a key role in photosynthesis regulation. Expression of this enzyme didn't show any change in the tolerant genotype GN‐3074 under drought stress, while it decreased in the sensitive genotype GN‐2032, Similarity, the expression of Rubisco didn't show any change in the GN‐3074, while the GN‐2032 experienced significant decrease in expression under drought stress condition (IF = 0.001). Yu stated that the abundance of Rubisco Activas enzyme in the soybean sensitive genotype under severe stress condition decreased 472 times while it increased in the tolerant genotype 67 times (Yu et al., 2016). In overall, our results demonstrated light reaction strongly affected by drought stress and proteins related to light reaction increased in abundance under stress compared with normal condition in GN‐3074 whereas in GN‐2032 genotype the photosynthetic proteins decreased in abundance under drought stress. Significant changing patterns of proteins in two soybean genotypes (GN‐3074‐drought tolerant) and (GN‐2032‐drought sensitive) revealed the processes that underlie the stay green and senescence trait in GN‐3074 and GN‐2032 genotypes, respectively. Stay‐green genotypes maintained normal/higher photosynthesis during drought stress because of delayed expression of senescence‐related genes (Lim et al., 2007). Bryant et al. (2018) in their study on molecular mechanisms of stay green in *Sorghum bicolor* under drought stress, observed high levels of photosynthesis proteins in stay‐green genotype and they suggested photosynthetic efficiency is protected and maintained under stressed conditions, which is a typical trait of the stay‐green phenotype (Bryant, 2018). Stay‐green is one of drought tolerance mechanisms of plant, in which leaf chlorophyll content and photosynthetic activity is maintained for longer despite drought stress conditions, it is a valuable agronomic trait in crop species (Borrell & Hammer, 2000; Harris et al., 2006). Previous study on wheat(Bazargani et al., 2011) and white clover (Wilson et al., 2002) reported decrease in abundance of photosynthetic proteins in senescing leaf. Based on our results, it seems that the drought‐tolerant genotype tries to protect sugar product activity by increasing or fixing the photosynthesis enzymes (stay green) under stress conditions, considering that it had a more stomata conductivity (Figure 2). It can be said tolerant genotype could have more gas exchanges by stomata conductivity protection and for this reason, enzyme expressions did not decrease, and their increased expression caused the production of more sugar (Tables 1 and 2).

**TABLE 1:** The corresponding induction factor (percent volume of spot in stress condition/percent volume of spot in well‐watered condition) of drought responsive proteins of soybean leaf identified using MS

Spot number	Ave GN3074 normal	Ave GN3074 Stress	Ave GN2032 normal	Ave GN2032 Stress	IF(GN−3074)	IF(GN−2032)	MW	PI
8	0.35	1.17	0.68	1.80	**3.32**	**2.64**	21.0	5.1
10	0.32	1.06	0.99	1.63	**3.29**	**1.66**	22.0	5.4
61	1.63	0.59	1.25	0.53	**0.36**	**0.42**	41.0	6.0
64	0.99	1.09	1.55	0.49	1.10	**0.31**	51.0	5.8
79	0.88	0.87	1.57	0.68	0.99	**0.43**	53.0	4.8
136	0.14	0.56	0.59	2.71	**4.10**	**4.62**	16.0	4.2
142	0.21	1.40	0.68	1.72	**6.80**	**2.53**	29.0	4.8
151	1.59	0.70	0.96	0.75	**0.44**	0.78	39.0	5.1
157	1.76	0.71	0.33	1.20	**0.40**	**3.61**	12.0	6.1
161	0.62	1.30	0.61	1.47	**2.09**	**2.39**	19.0	5.3
182	1.13	0.54	1.30	1.03	**0.48**	0.79	26.0	5.3
241	0.73	1.77	0.97	0.52	**2.41**	0.54	46.0	5.7
292	1.38	1.17	1.45	0.00	0.85	**0.00**	34.0	4.9
294	1.42	0.04	2.38	0.09	**0.02**	**0.04**	54.0	6.6
352	0.84	1.52	1.12	0.37	**1.81**	**0.33**	27.0	4.7
355	1.25	0.86	1.89	0.00	0.69	**0.00**	35.0	4.6
376	3.90	0.09	0.03	0.02	**0.02**	0.76	15.0	5.6
419	0.35	0.96	1.05	1.64	**2.79**	**1.56**	29.0	5.1
459	3.97	0.04	0.00	0.00	**0.01**	0.75	32.0	6.2
487	0.73	0.72	0.53	2.08	0.99	**3.93**	22.0	4.9

Bold IFs represent change statistically significant in at least one variety in response to drought stress compared to well‐watered. Spots were concluded to be significantly up‐ or down‐regulated when *p* < .05.

**TABLE 2:** Details of 20 identified proteins under drought stress in leaf tissue of soybean

Spot number	Name of protein	Function	Accession number
79	Portable Ca binding Protein CML 33	Calmodulin	Q9SRP4
241	Ferredoxin ‐NDP reductase	Electron transport chain	Q9AWB2
294	trypsin inhibitor	Enhance defense mechanism	P01070
292	Chlorophyll a‐b binding protein of LHCI	Photosynthesis‐Light‐harvesting	P12471
142	Chlorophyll a‐b binding protein	Photosynthesis	C6TD73
352	2cys‐peroxiredoxin BS1‐like	Anti‐oxidant defense	A0A0B2RB28
419	Ribolus bisposphate carboxylase small chain PW9	Photosynthesis	P26667
8	Ascorbate Peroxidase	Anti‐oxidant defense	Q43758
10	Amino‐methyl Transferase	Methylation Amino acids	A0A0R0IB61
61	S‐Adenosyl Methionine Synthetase	Amino acid biosynthesis	C6TNJ3
64	RUBISCO Activase	Photosynthesis	D4N5G3
136	Heat‐shock protein	Defense Protein	A0A0B2QPZ4
151	Quinone oxidoreductase‐like protein	Anti‐oxidant defense	A0A0B2QPS9
157	Glyceraldehyde 3‐phosphate de hydrogenas subunit α	Calvin cycle	Q38IX1
161	Eukaryotic translation initiation factor 5A (eIF5A)	Translation factor	C6ZHS4
182	Ferritin	Fe storage in chloroplast	P19976
355	RUBISCO	Photosynthesis	A0A0F6R3U4
376	Nucleoside diphosphate kinase	Phosphor movements	Q39839
459	Putative r40c1 protein	Phosphoprotein	C6TNS9
487	Glyceraldehyde 3‐phosphate de hydrogenas subunit β	Calvin cycle	Q38IX0

Five identified proteins included defense proteins, of which three were anti‐oxidant enzymes. One of them was defense protein and another was used for activation of anti‐oxidant defense. Three proteins were involved in oxidative stress response protein (Tables 1 and 2), Ascorbate peroxidase (spot 8) expression increased in both genotypes severely. However, 2cys‐peroxiredoxin BS1‐like (spot 352) increased only in the GN‐3074 but decreased in the GN‐2032. The expression of quinone oxidoreductase‐like protein (spot 151) didn't show any change in the sensitive genotype but increased in the tolerant genotype (Tables 1 and 2). Generally, abiotic stresses (e.g., drought) can increase the production of reactive oxygen species (ROS) resulting the breakdown of photosystems due to senescence (by downregulation of photosynthesis‐related protein) results in disruption of normal electron flow through the light‐harvesting complexes causing oxidative damage, therefore plant cells in this condition require mechanisms to detoxify excess ROS. Therefore, the susceptibility of genotype GN‐2032 to drought stress can be explained by low abundance of both photosynthetic and oxidative stress defense proteins. In contrast in tolerant genotype GN‐3074 despite of high abundance of photosynthetic proteins, two oxidative stress defense proteins showed increase in abundance to induce more drought stress tolerance.

The trypsin inhibitor protein as a defense protein decreased in both genotypes under drought condition, of course with its more reduction in tolerant genotype GN‐3074 as compare with GN‐2032 (Tables 1 and 2). The trypsin inhibitor and dehydroascorbate reductase are able to decrease the dehydroascorbate activity (Inzé & Van Montagu, 1995). Therefore, a decrease in trypsin inhibitor activity results in an increase in dehydroascorbat activity. Consequently, a decrease in trypsin inhibitor expression led to an increase in the abundance of ROS harvesting protein. Proteinase inhibitors are part of a defense mechanism that depends on Jasmonic acid and accumulation on injury, plants damage, and pathogens (Farmer et al., 2003). Trypsin inhibitor decreased in both genotypes and this may be a reason to enhance defense mechanisms by increase in defense protein synthesis.

Heat shock Protein (HSP) (spot 136) expression increased greatly in both genotypes under drought condition (Tables 1 and 2). Molecular chaperones/heat‐shock proteins (HSPs) are responsible for protein stabilization, proper folding, assembly, and translocation under both optimum and adverse growth conditions (Hossain et al., 2013).

The results showed Ferritin expression decreased significantly in the tolerant genotype under drought stress condition (IF = 0.48), but did not show any significant change in the sensitive genotype (IF = 0.79). Previous studies have also revealed that plant ferritins protect cells against oxidative damage (Ravet et al., 2009). Tolerant genotype GN‐3047 because of high abundance of photosynthetic proteins as a stay‐green phenotype can be had low oxidative damage, while sensitive genotype GN‐2032 despite of low photosynthesis which can be caused more oxidative damage showed no change for ferritin protein expression to protect it from ROS damage and even cell death.

Glyceraldehyde 3‐phosphate de hydrogenase subunit α and b (spots 157 and 487) are two proteins related to carbon metabolism or energy metabolism which up‐regulated in sensitive genotype GN‐2032, in contrast, they showed decrease in abundance/no change in tolerant genotype GN‐3074. A reduction in metabolism rate is likely a major factor which induce delayed leaf senescence (stay‐green) in plants (Lim et al., 2003). Therefore, the reduction and increase in expression of Glyceraldehyde 3‐phosphate de hydrogenase in GN‐3074 and GN‐2032 can be confirmed mechanism of stay‐green and senescence as a reason of their drought tolerance and susceptibility.

The other identified protein was Amino‐Methyl Transferase (Tables 1 and 2). This enzyme expression increased (IF = 3.29) in the GN‐3074 under drought stress, and also increased a little in the genotype GN‐2032 (IF = 1.6). Amino‐methyl transferase (AMT) and Glycine d hydrogenase abundance decreased in the sensitive genotype under drought conditions, but it was stable or increased in the tolerant genotype (Zhao et al., 2011).

In the group of signal transduction proteins, we identified Portable Ca^+^ binding Protein CML 33. The expression of this protein didn't show any change in the tolerant genotype but decreased in the sensitive genotype (Tables 1 and 2). This protein plays an important role in signal transduction and response to drought stress. It is possible, the stress signals trigger transient changes in the cytosolic Ca^2+^ level which acts as a second messenger. Ca^2+^ sensors in turn transmit and activate the signaling pathways for downstream stress responses(Xiong & Zhu, 2002). The expression of the soybean CaM (GmCaM4) activated R2R3, which in turn, up‐regulated several genes, including P5CS which involved in response to drought stress (encoding a proline anabolic enzyme) (Yoo et al., 2005). Therefore, the susceptivity of genotypeGN‐2032 can be explained by low abundance of CaM under drought stress, whereas genotype GN‐3074 could fix the level of this protein expression, and it was able to transduce drought stress signals. It had a better reaction under stress condition and was able to accumulate proline in its cells. These consequences activate the phosphoprotein cascade, which ultimately activates the transcription of ABA biosynthesis precursors and finally synthesizes ABA that aids in stomatal closure to reduce transpiration levels, CO_2_ assimilation, and photosynthesis for the sake of survival of the plant under adverse environmental conditions (Tuteja, 2007).

Two proteins identified in the group phosphorus transduction, which play several roles in phosphorus transduction in cells. One of them is a nucleoside diphosphate kinase enzyme which plays a role in energy metabolism. This enzyme plays a role in ATP production during *Glycolysis* metabolism. The expression of this enzyme didn't show significant decrease in GN‐2032 under drought stress, while decreased in GN‐3074 (Tables 1 and 2). Yu et al. (2016) reported that drought stress can increase the expression of these enzymes in the tolerant genotype under severe drought stress. This enzyme is a respiration enzyme and is involved in *Glycolysis(*Yu et al., 2016 *)*. However, in this study, the expression of this enzyme decreases in the tolerant genotype and remains without any change in the sensitive genotype. Another protein found in this group was Putative r40c1 protein. This protein has a role in phosphorus transduction. This protein decreased in the GN3074 but had no significant change in the GN‐2032. The expression of Adenosyl Methionine synthesis decreases in both genotypes. Adenosyl methionine synthesis is involved in ethylene synthesis. This enzyme has a role in numerous methyl transduction reactions as a methyl group donor and plays a role in polyamine and ethylene biosynthesis (Peleman et al., 1989). Another role of this molecule is the regulation of methionine synthesis and other amino acids derived from aspartate that can participate during the other important protein synthesis.

## 4 CONCLUSIONS

Proteomics analysis was applied in our study to elucidate the molecular mechanisms underlying soybean response to drought stress. The proteome pattern of two soybean genotypes GN‐3074 (drought tolerant) and GN‐2032 (drought‐sensitive) was compared under drought stress condition. The results herein presented, reflects variations in molecular levels and change their proteins expression that soybean plants apply in adapting to the drought stress environment. The differential abundance of proteins in two genotypes may suggest that drought‐tolerant of soybean genotype GN‐3074 is due to its efficient photosynthesis which is increased compare to sensitive genotype GN‐2032 and also is due to its higher oxidative stress defense response as a protective mechanism to avoid generation of ROS. Taken together, these results provide new insights of stay‐green mechanism in tolerance of soybean to drought stress through regulation of proteins associated with photosynthetic machinery and also managing ROS scavenging, oxidative damage to help be sustain under drought stress. This information suggests that these proteins may be important targets for soybean breeding programs to enhance plant tolerance during vegetative stage under drought condition, but it is necessary to more studies regarding plant response to drought stress, in this regard, we suggest several strategies, (a) Enhancement of stay‐green proteins expression pattern, (b) mapping population derived from cross of GN‐3047 and GN‐2032 genotypes to understand genetic basis of proteins related to stay‐green mechanism and finally identification important proteins as markers which can be applied for breeding program.

## INFORMED CONSENT

Written informed consent was obtained from all study participants.

## CONFLICT OF INTEREST

The authors declare that they do not have any conflict of interest.

## ETHICAL APPROVAL

This study does not involve any human or animal testing.

## Data Availability

All authors confirm that the data supporting the findings of this study are available within the article.
